# Methotrexate inhibits glucocorticoids-induced osteoclastogenesis via activating IFN-γR/STAT1 pathway in the treatment of rheumatoid arthritis

**DOI:** 10.1136/rmdopen-2024-004886

**Published:** 2024-11-07

**Authors:** Yao Teng, Haifeng Yin, Ruizhi Feng, Lijuan Jiang, Wenlin Qiu, Xiaoru Duan, Xuefei Wang, Guo-Min Deng

**Affiliations:** 1 Department of Rheumatology and Immunology, Union Hospital, Tongji Medical College, Huazhong University of Science and Technology, Wuhan, Hubei, China

**Keywords:** Arthritis, Methotrexate, Osteoporosis, Inflammation, Glucocorticoids

## Abstract

**Objectives:**

Rheumatoid arthritis (RA) is a chronic autoimmune disease characterised by the synovitis and bone erosion. The combination therapy of glucocorticoids (GCs) and methotrexate (MTX) is recommended in early RA management, although the precise underlying mechanism of action remains unclear. This study is aimed to clarify the mechanism of MTX in combined with GC in treating RA.

**Methods:**

GC-induced osteoporosis (GIOP) mouse model was used to investigate the bone-protective role of MTX. Lipopolysaccharide-induced arthritis mouse model was used to evaluate the anti-inflammatory effects of GCs and MTX. Functional role of MTX on osteoclastogenesis was assessed by trap staining and micro-computer tomography. Western blot, RT-qPCR and coimmunoprecipitation were used to explore the underlying mechanisms.

**Results:**

We demonstrate that GCs, but not MTX, rapidly inhibited synovitis in arthritis model. MTX treatment was observed to inhibit osteoclastogenesis induced by GC in vitro and mitigate bone loss attributed by GIOP. GCs were found to augment the interaction between the membrane GC receptor (mGR) and signal transducer and activator of transcription 1 (STAT1), leading to the suppression of IFN-γR/STAT1 signalling pathways. Interestingly, MTX was found to inhibit osteoclastogenesis induced by GCs through the enhancement of the A2AR and IFN-γR interaction, thereby activating the IFN-γR/STAT1 signalling cascade. Consequently, this process results in a reduction in the mGR and STAT1 interaction.

**Conclusions:**

Our study provides compelling evidence that MTX can make GCs effectively to suppress synovitis and reduce bone loss induced by GCs. This sheds light on the potential mechanistic insights underlying the efficacy of GCs in conjunction with MTX for treating RA.

**WHAT IS ALREADY KNOWN ON THIS TOPIC**

- In the clinical practice, the combination therapy of glucocorticoids (GCs) and methotrexate (MTX) is strongly recommended in early rheumatoid arthritis (RA) management.
- GCs can rapidly suppress acute inflammation.
- GCs promote osteoclast formation by binding to membrane GC receptor (mGR).

**WHAT THIS STUDY ADDS**

- MTX reduces side effects of bone loss induced by GCs in the treatment of RA.
- MTX was discovered to inhibit osteoclastogenesis induced by GCs by enhancing the A2AR and IFN-γR interaction, subsequently activating the IFN-γR/signal transducer and activator of transcription 1 (STAT1) signalling cascade. As a result, this process diminishes the mGR and STAT1 interaction.

**HOW THIS STUDY MIGHT AFFECT RESEARCH, PRACTICE OR POLICY**

- Our work will impact our understanding of the potential mechanistic insights underlying the effectiveness of the combination of GCs with MTX in the treatment of RA.

## Introduction

 Rheumatoid arthritis (RA) is a systemic autoimmune disorder characterised by synovitis and bone erosion,^1 2^ impacting the physical function and life quality of approximately 0.5%–1.0% of the global population.^3^ Methotrexate (MTX) in combination with glucocorticoids (GCs) represents the primary therapeutic approach for early RA management, effectively reducing inflammation and bone damage.^4 5^ However, the precise underlying mechanism of action remains inadequately understood.

GCs are the preferred treatment during the acute phase of RA, particularly when patients exhibit extra-articular manifestations (such as lung, eye, nervous system) or accompanied with other immune diseases. Prolonged GC therapy or excessive endogenous GC levels can lead to irreversible adverse effects such as osteoporosis, exacerbating bone degeneration in RA.^1^ GCs exert their multiple actions via genomic and non-genomic pathways. The anti-inflammatory and immunosuppressive properties of GCs are primarily mediated through binding to cytosolic GC receptors (GR), translocating to the nucleus and inhibiting transcription of inflammatory genes, known as trans-repression.^6^

MTX has been recommended as the initial RA treatment according to the EULAR recommendations and the American College of Rheumatology guidelines.^7 8^ MTX has demonstrated a bone-protective role in RA.^9^ Nonetheless, some patients experience high disease activity characterised by joint destruction or systemic symptoms, necessitating the combination therapy of GCs and MTX as the preferred option to rapidly alleviate inflammation and mitigate bone damage. GC-induced osteoporosis (GIOP) results from reduced bone formation by osteoblasts and increased bone resorption by osteoclasts (OCs),^10 11^ which derive from myelomonocytic precursor cells/macrophage lineage. Aberrant OC-mediated bone resorption contributes to bone loss.^12^ GC-induced osteoclastogenesis primarily involving membrane GC receptor (mGR) activation.^13^ MTX has been shown to inhibit OC formation induced by receptor activator of nuclear factor κB ligand (RANKL), yet its impact on osteoclastogenesis induced by GCs remains unclear. Furthermore, the underlying mechanism of MTX in conjunction with GCs for RA treatment remain elusive.

Hence, this study aims to elucidate the mechanistic insights behind the combined therapy of MTX and GCs in RA treatment and identify potential targets for bone erosion mitigation.

## Methods

### Arthritis inflammation mouse model

C57BL/6 mice aged 8 weeks were injected intra-articularly with 50 ng lipopolysaccharide (LPS) to induce arthritis. These mice were divided into the Ctrl, LPS, L+M (1 mg/kg intraperitoneal injection of MTX for 3 days), L+D (5 mg/kg intraperitoneal injection of dexamethosone (DXM) for 3 days) and L+D+M (intraperitoneal injection of 1 mg/kg MTX and 5 mg/kg DXM) groups, each group have 10 mice. Three days later, mice were then sacrificed. Femurs were treated with formalin and embedded in paraffin following standard procedure.

### GIOP murine model

GIOP mouse model was established through intraperitoneally injecting with 5 mg/kg DXM daily for 28 days. Eight-week-old male C57BL/6 mice were divided into the Ctrl, MTX, DXM and D+M groups. Ctrl group was injected with normal saline. MTX group was injected with MTX 1 mg/kg for every 3 days. DXM group was injected with 5 mg/kg/day dexamethasone daily. D+M group treated with DXM and MTX. Femurs were harvested on day 29 and then treated with formalin. Next, the femurs were decalcified for 30 days.

Other methods are provided in online supplemental materials.

## Results

### GCs but not MTX can inhibit acute inflammation

**Figure 1:**
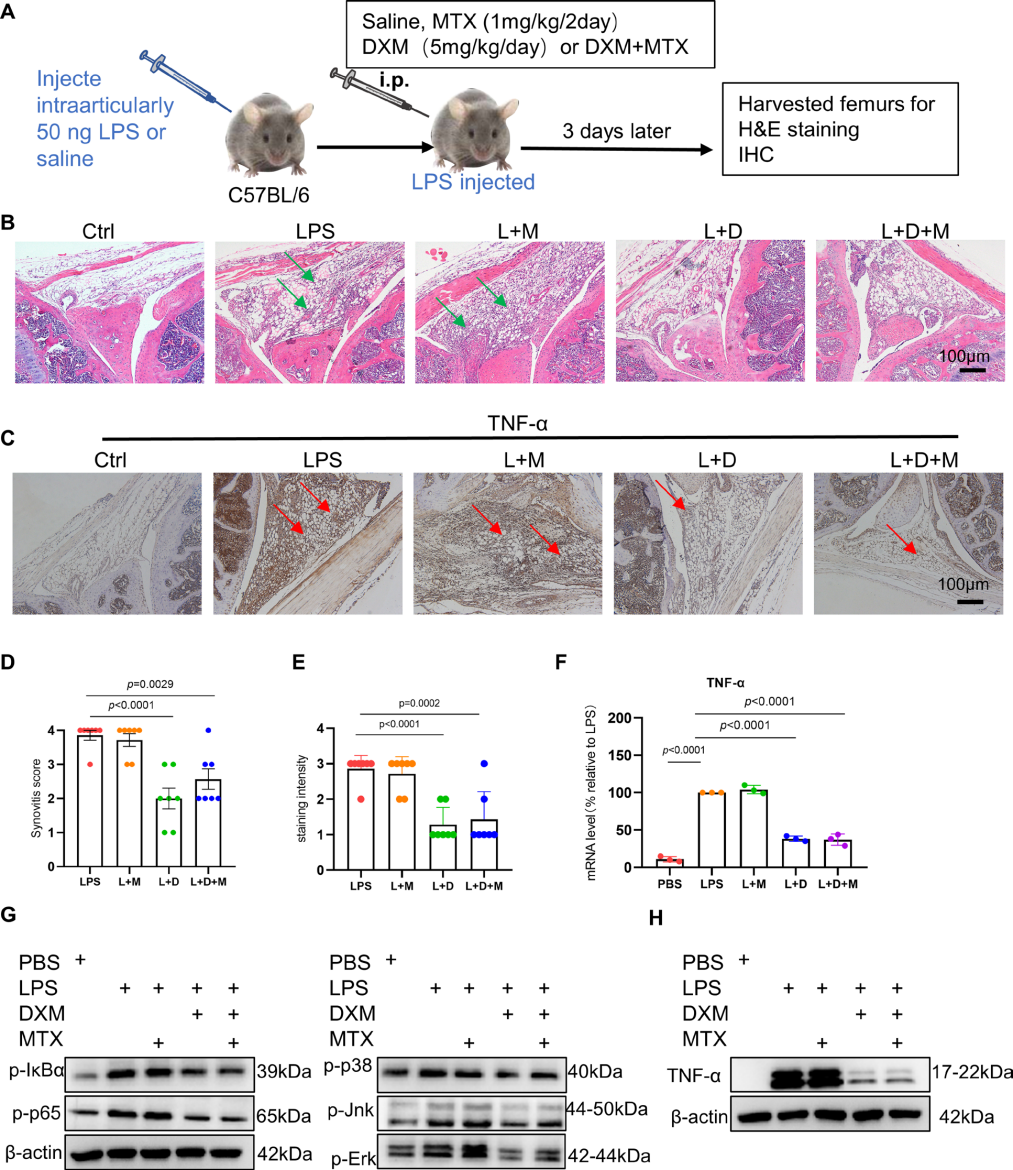
The effects of GCs and MTX in LPS-induced acute inflammation. (**A**) Schematic representation of established process of the arthritis inflammation mouse model. (**B,C**) Representative photomicrographs of H&E staining (**B**) and immunohistochemistry (IHC) staining (**C**) of TNF-α of knee joints showing Ctrl, LPS (50 ng), LPS (50 ng)+MTX (1 mg/kg), LPS (50 ng)+DXM (5 mg/kg), LPS (50 ng)+DXM (5 mg/kg)+MTX (1 mg/kg). n=7 per group; green arrows indicate inflammation of synovial tissue, red arrows indicate TNF-α positive areas of synovial tissue. Scale bar: 100 µm. (**D**) Quantification of the histological analysis of synovitis (n=7 per group). (**E**) Quantification of IHC staining of TNF-α. Data are shown as means±SD from two separate experiments. Bone marrow-derived macrophages (BMDMs) were stimulated by LPS (100 ng/mL) in the presence of phosphate-buffered saline, DXM (10 µm), MTX (5 um) or DXM+MTX treatment and treated with DXM (10 µm) or MTX (5 µm), which were divided into PBS, LPS, L+M, L+D and L+D+M group. L represents LPS, D represents DXM, M represents MTX. (**F**) The mRNA level of TNF-α in above four group cells by RT-qPCR. (**G,H**) Representative Western blotting images of nuclear factor kappa-B and mitogen-activated protein kinase signalling pathway protein levels (**G**) and TNF-α expression (**H**) in above four group cells. In (**D**), (**E**), (**F**), data were analysed using a one-way analysis of variance, post hoc Dunnett’s test. Data are shown as the means±SD of two separate experiments. DXM, dexamethosone; GCs, glucocorticoids; LPS, lipopolysaccharide; MTX, methotrexate.

In clinical practice, the combination of MTX and GCs emerges as the preferred therapeutic strategy for patients with early-stage RA. It is known that GCs can rapidly inhibit acute inflammation,^13^ but it is not clear whether MTX can rapidly inhibit acute inflammation, thus we performed experiments to determine whether MTX rapidly inhibits arthritis. To this aim, we used a mouse model of LPS-induced arthritis inflammation as depicted in figure 1A, which has been shown that TNF-α/TNFR1 plays a critical role.^14^ We found that severity of LPS-induced arthritis was significantly decreased in mice treated with dexamethosone (DXM) and mice with combined therapy of DXM and MTX, but not in mice treated with MTX compared with saline treatment (figure 1B–D); we also found that severity of arthritis was similar in mice treated with DXM and mice with combined therapy of DXM and MTX. These results suggest that GCs but not MTX can inhibit acute arthritis (figure 1B,D).

To know why GCs but MTX can inhibit acute inflammation, we did immunohistochemistry staining in arthritis induced by LPS with GCs treatment and MTX treatment. We found that there was a large amount of TNF-α in arthritis induced by LPS in mice with MTX treatment and with saline treatment, but much less TNF-α in arthritis induced by LPS in mice with DXM and combined therapy of DXM and MTX. These data indicate that GCs can inhibit acute arthritis through reducing production of TNF-α, MTX cannot reduce acute arthritis due to lacking of reducing the production of TNF-α (figure 1C,E).

LPS can stimulate inflammatory signalling pathways involving nuclear factor kappa-B (NF-κB) and mitogen-activated protein kinase (MAPK), resulting in the excessive production of TNF-α.^15 16^ Subsequently, we investigated whether DXM and MTX modulate NF-κB and MAPK activation induced by LPS in vitro Western blotting results showed that DXM effectively suppressed NF-κB and MAPK activation, leading to reduced TNF-α release (figure 1F–H). On the contrast, MTX failed to inhibit NF-κB and MAPK activation, as well as the release of TNF-α based on RT-qPCR and western blotting data (figure 1F–H). Taken together, these results validate that DXM but not MTX has the potent and rapid anti-inflammatory effects.

### MTX inhibit osteoclastogenesis induced by DXM in vitro

**Figure 2:**
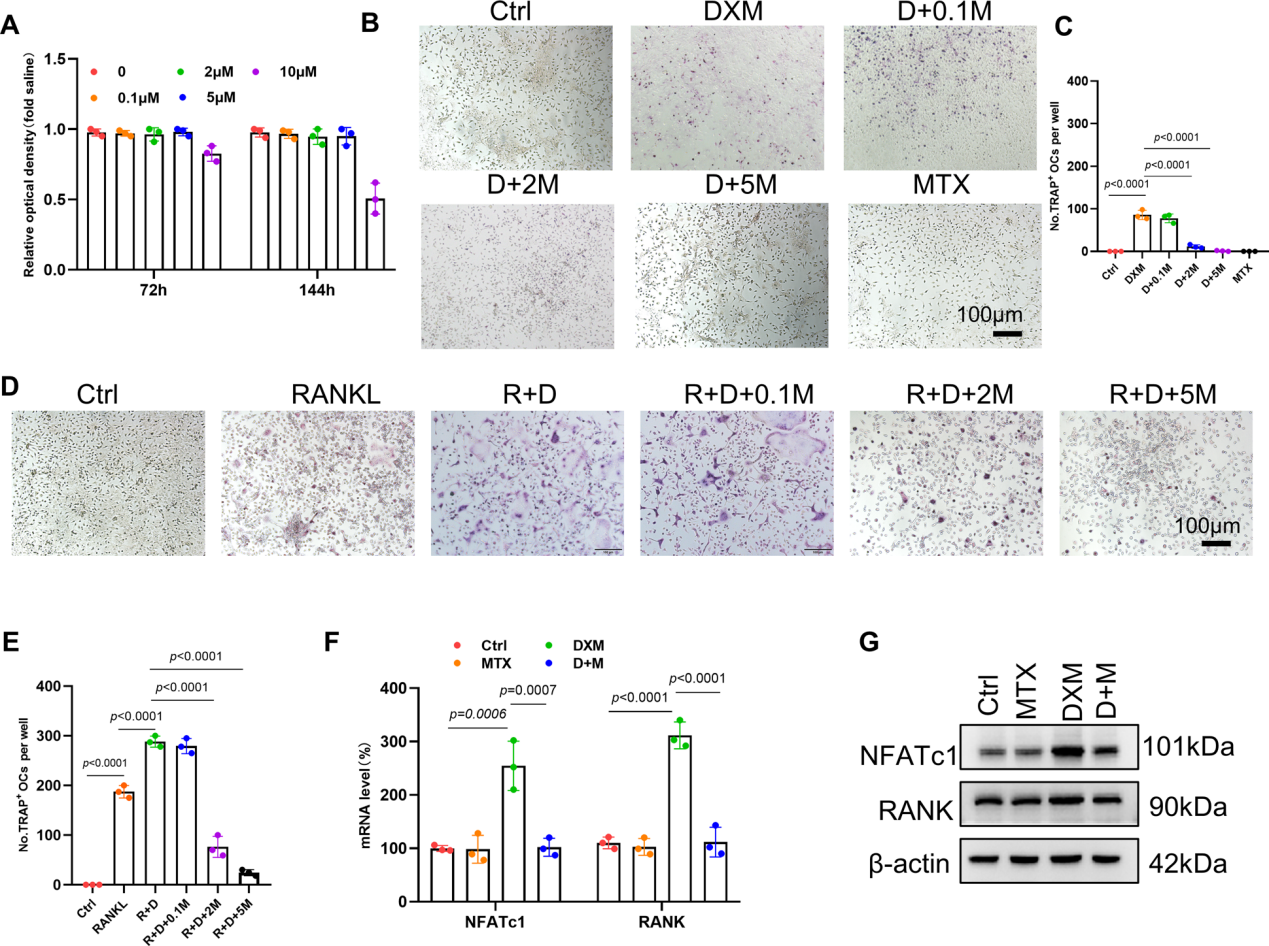
The role of MTX in DXM-induced osteoclastogenesis. (**A**) Viability of bone marrow-derived macrophages (BMDMs) treated with various dose of MTX were evaluated via CCK8 assay at 72 hours and 144 hours. (**B,C**) Representative images (**B**) and quantification (**C**) of TRAP-positive osteoclasts cultured with DXM in the presence or absence of various doses of MTX. (**D**) Representative images of TRAP-positive osteoclasts in BMDMs cultured with DXM and RANKL and/or MTX for 3 days. Scale bar: 100 µm. (**E**) Quantification of TRAP-positive osteoclasts cultured with DXM in the presence or absence of various doses of MTX. (**F,G**) BMDMs treated with DXM and PBS in the presence or absence of MTX. The mRNA level (**F**) and the protein level (**G**) of NFATc1 and RANK in BMDMs from four group cells were evaluated by RT-qPCR or western blot. In (**E**), (**F**), data were analysed using a one-way analysis of variance, post hoc Tukey’s test. Data are shown as the means±SD of three separate experiments. DXM, dexamethosone; MTX, methotrexate; NFATc1, nuclear factor of activated T cells 1; RANK, receptor activator of nuclear factor κB.

Accumulating studies indicated that MTX inhibits osteoclastogenesis stimulated by RANKL.^9 17 18^ Thus, we investigated the effects of MTX on osteoclastogenesis induced by DXM in vitro. We first evaluated the effect of MTX on viability of OC precursor cells for 72 and 144 hours to determine dose toxicity. According to cell counting kit-8 results, MTX exhibited no cytotoxic effects on viability of OC precursor cells at doses below 5, 2 or 0.1 µM, respectively (figure 2A). Then we determined the effect of MTX on osteoclastogenesis induced by DXM or DXM combined with RANKL. As shown in figure 2B,C, MTX significantly inhibited the formation of TRAP-positive OCs at a dose-dependent manner. Furthermore, MTX significantly decreased the number TRAP-positive multinucleated OCs induced by DXM and RANKL at a dose-dependent manner (figure 2D,E). In our previous study,^13^ we found that DXM increased protein and mRNA level of nuclear factor of activated T cells 1 (NFATc1) and RANK, which are crucial factors in osteoclastogenesis.^19^ As shown in figure 2F,G, RT-qPCR and Western blotting results displayed that the addition of MTX could obviously reduce the mRNA and protein level of NFATc1 and RANK compared with DXM group.

On the other hand, we investigated whether MTX affected the inhibitory effect of GCs on osteoblasts. RT-qPCR results showed that MTX did not reverse the inhibitory effect of DXM on mRNA expression of osteoblastogenesis-related genes, including Bglap2, OPN, alkaline phosphatase (ALP) and RUNX2 (online supplemental figure 1A). ALP results showed that MTX did not rescue the number of the decreased ALP-positive osteoblasts caused by DXM (online supplemental figure 1B,C). Taken together, these findings highlight the inhibitory role of MTX on DXM-induced osteoclastogenesis without interfering with the osteoblast-suppressive effects of GCs

### MTX significantly ameliorates GIOP

**Figure 3:**
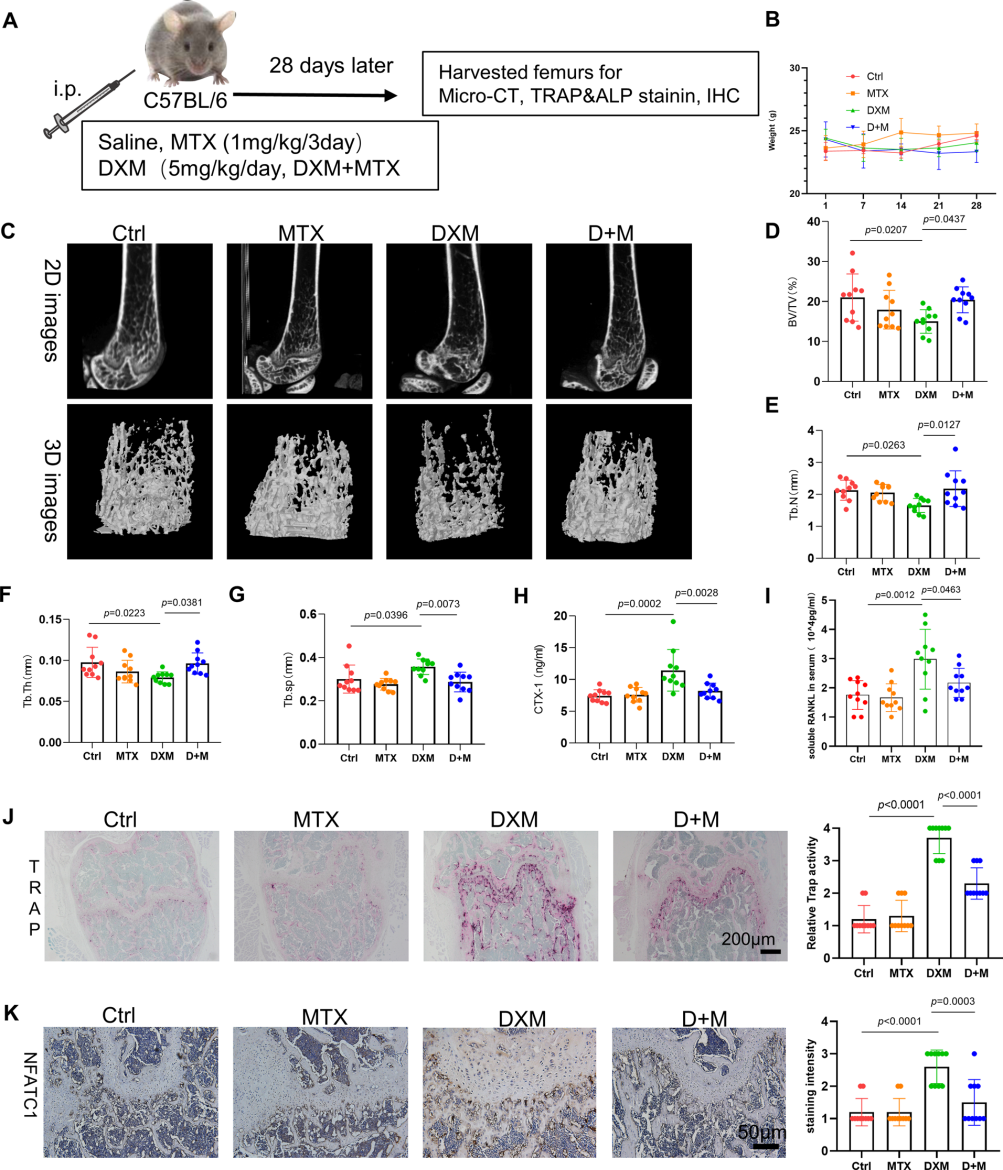
The role of MTX in glucocorticoids induced osteoporosis (GIOP). (**A**) Schematic representation of established process of the GIOP mouse model. (**B**) Body weight of four group mice detected every 7 days. (**C**) Representative 3D and 2D reconstruction images of femur and trabecula of mouse showing Ctrl, MTX (1 mg/kg), DXM (5 mg/kg), D (DXM 5 mg/kg)+M (MTX,1mg/kg); n=10 per group. (**D**) Statistical analysis of the ratio of bone volume to tissue volume (BV/TV). (**E**) Statistical analysis of trabecular number (Tb.N). (**F**) Statistical analysis of trabecular number trabecular thickness (Tb.th). (**G**) Statistical analysis of trabecular separation (Tb.Sp). (**H,I**) The level of serum cross-linked carboxy-terminal telopeptide of type I collagen (CTX-I) (**H**) and RANKL (**I**) of mice detected by ELISA. (**J**) Representative images and scores of TRAP staining of the femur; scale bar: 200 µm. (**K**) Representative images and scores of IHC of nuclear factor of activated T cells 1 of the femur; scale bar: 50 µm. In (**D**), (**E**), (**F**), (**G**), (**H**), (**I**), (**J**), (**K**), data were analysed using a one-way analysis of variance, post hoc Tukey’s test. Data are shown as means±SD from two separate experiments. DXM, dexamethosone; IHC, immunohistochemical; MTX, methotrexate; RANKL, receptor activator of nuclear factor κB ligand.

We have observed that MTX could inhibit DXM-induced osteoclastogenesis in vitro. Although GCs are commonly employed to rapidly achieve inflammation remission in RA, they can also lead to secondary osteoporosis.^20 21^ In this study, we assessed the impact of MTX in a mouse model of DXM-induced osteoporosis following the experimental schedule outlined in figure 3A. We found no significant difference in the weight changes of mice among these four groups (figure 3B). After a 28-day period, micro-CT imaging was used to assess osteoporosis in the femurs of the four groups. The 2D and 3D images depicting trabecular bone loss showed evident bone loss in the cortical bone of mice treated with DXM compared with mice treated with PBS, whereas bone loss was notably attenuated in mice with combined therapy of MTX and DXM compared with mice with DXM (figure 3C). Furthermore, the bone volume/tissue volume ratio was significantly reduced in the group receiving DXM along with MTX as compared with the DXM-only group (figure 3D). Trabecular number (Tb.N) and trabecular thickness (Tb.th) exhibited marked decrease in the DXM group relative to the control group, with further enhancements observed in the group receiving DXM combined with MTX (figure 3E,F). Conversely, trabecular separation (Tb.Sp) was significantly increased in the DXM group compared with the D+M group (figure 3G). Additionally, we further evaluated osteoporosis in these mice by examining the serum level of cross-linked carboxy-terminal telopeptide of type I collagen (CTX-I) and RANKL, which was an indicator of DXM-induced osteoporosis.^22^ ELISA analysis revealed that CTX-I level and RANKL level were elevated in mice treated with DXM than the control mice, while it was effectively decreased in mice with combined therapy of DXM and MTX compared with mice with DXM treatment (figure 3H,I).

Subsequently, the mechanism underlying the ameliorative effects of MTX on GIOP was explored. TRAP staining and ALP staining were employed to evaluate the impact of MTX on osteoclastogenesis and osteoblastogenesis induced by DXM, respectively. TRAP staining analysis showed that osteoclastogenesis of mice with combined therapy of DXM and MTX was notably reduced than these mice treated with DXM (figure 3J). Given that NFATc1 serves as a key marker gene for OCs,^23^ NFATc1 expression was evaluated through immunohistochemical (IHC) staining. IHC results showed that NFATc1 expression was upregulated in the DXM group compared with the control, which was markedly diminished in mice with combined therapy of MTX and DXM (figure 3K). ALP staining results showed that ALP staining results showed that osteoblastgenesis was significantly reduced in mice treated with DXM compared with the control mice, but it was similar in mice with combined therapy of MTX and DXM (online supplemental figure 1D,E). Collectively, MTX appears to play a bone-protective role in GIOP by primarily inhibiting osteoclastogenesis rather than enhancing osteoblastogenesis.

### MTX does not influence on nuclear translocation and expression of GR

**Figure 4:**
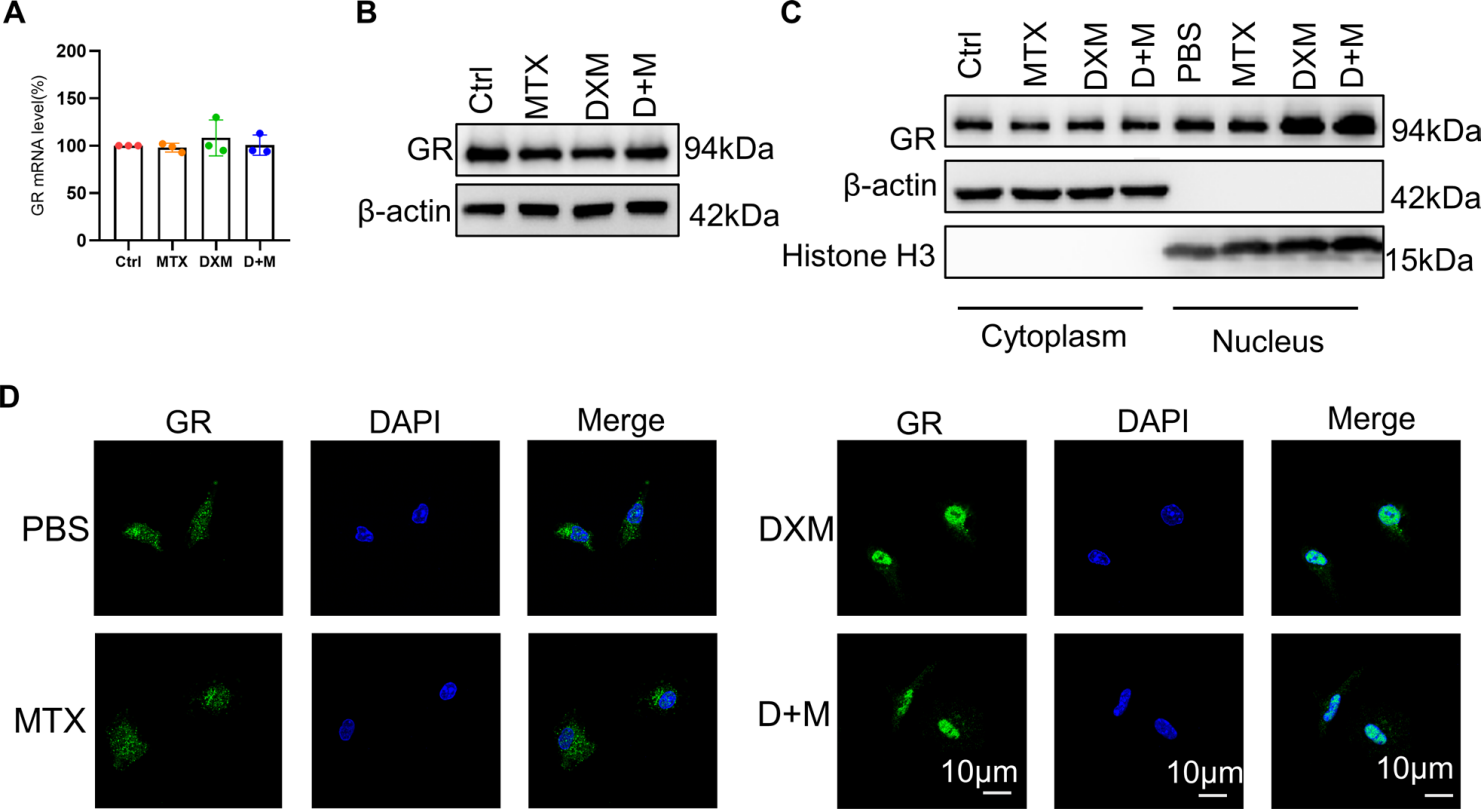
The role of MTX in DXM-induced nucleus translocation and expression of GR. (**A,B**) The mRNA level (**A**) and protein level (**B**) of GR in bone marrow-derived macrophages (BMDMs) showing Ctrl, MTX, DXM, D+M were evaluated by RT-qPCR or Western blotting (WB). D represents DXM, M represents MTX. (**C**) Protein level of GR in the nucleus and cytoplasm in BMDMs showing Ctrl, MTX, DXM, D+M were evaluated by WB. (**D**) Immunofluorescence images of GR location in BMDMs showing Ctrl, MTX, DXM, D+M; scale bar: 10 µm. In (**A**), data were analysed using data were analysed using a one-way analysis of variance, post hoc Tukey’s test. Data are shown as means±SD from three separate experiments. DXM, dexamethosone; GR, glucocorticoids receptor; MTX, methotrexate.

Since the GCs exerts its anti-inflammatory role via binding to GR and inducing GR nuclear translocation, leading to the inactivation of pro-inflammatory proteins,^6^ we examined whether MTX influenced the nuclear translocation and expression of GR. We found that MTX showed no significant impact on the mRNA and protein level of GR (figure 4A,B). Nucleocytoplasmic separation assay and immunofluorescence staining results showed that DXM induced GR translocation into the nucleus, while the effect was not reversed by MTX (figure 4C,D). Taken together, these findings confirmed that MTX does not modulate DXM-induced GR nuclear translocation or GR expression, providing insight into why MTX does not influence the anti-inflammatory effects of GCs.

### MTX decreases the interaction of mGR and signal transducer and activator of transcription 1

**Figure 5:**
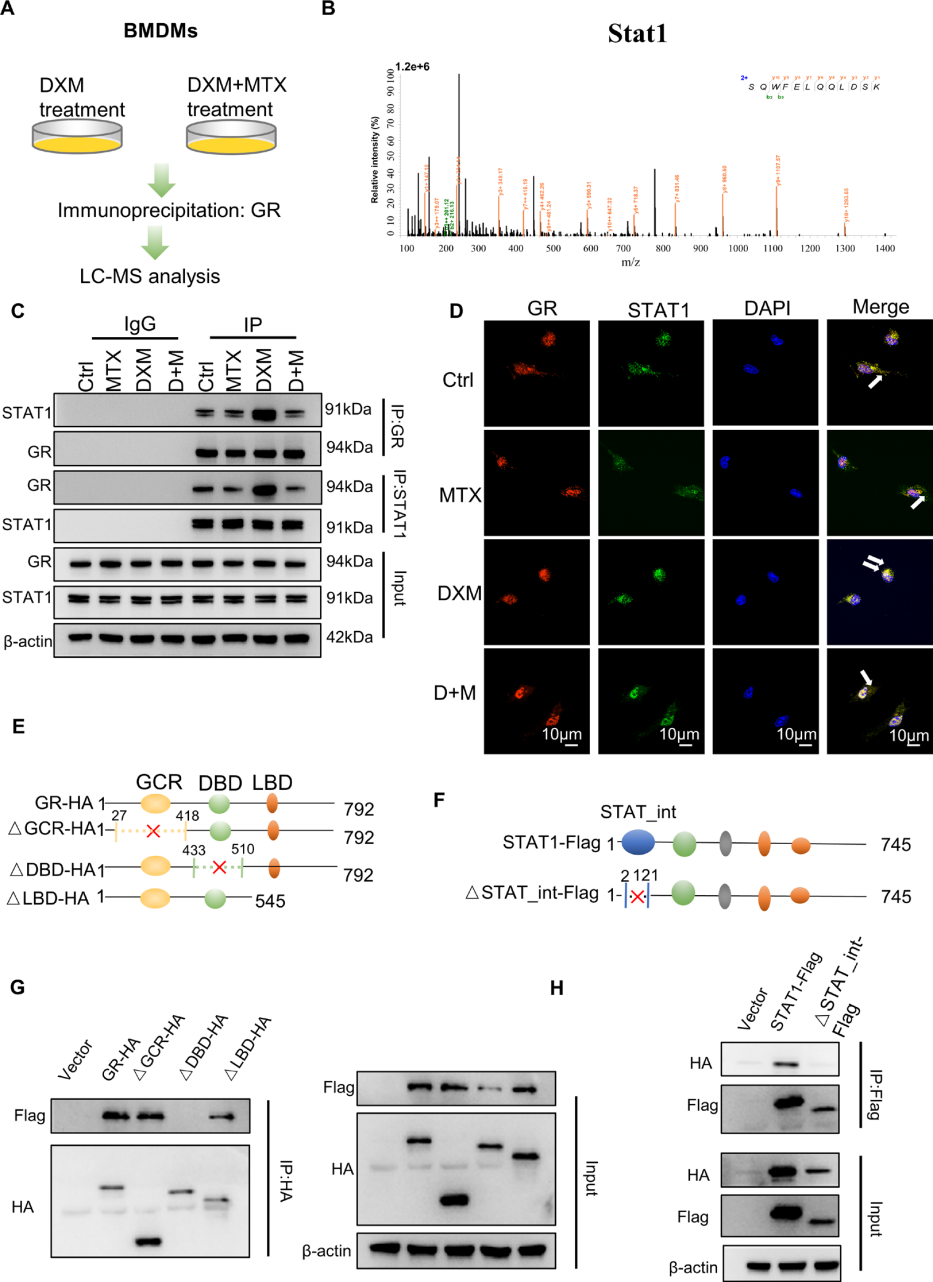
The interaction of mGR and STAT1. (**A**) Extracts of BMDMs cells treated with bovine serum albumin-conjugated DXM (DXM-BSA), or in the presence and absence of for 24 hours, the cells were immunoprecipitated with an anti-GR antibody. Eluted proteins were identified by LC-MS analysis. (**B**) STAT1 peptide fragment was precipitated with GR antibody by mass spectrometry (MS). (**C**) Extract of BMDMs treated with DXM-BSA, MTX or PBS for 24 hours was immunoprecipitated with IgG, GR and STAT1 antibodies. (**D**) Confocal microscopy was used to observe colocalisation of GR and STAT1 in BMDMs treated with DXM-BSA, MTX or PBS for 30 min, white arrows indicate the yellow area representing interaction of GR and STAT1 on the membrane; scale bar: 10 µm. (**E**) The full-length GR (GR-HA) was divided into three functional truncations (△27-418aa named △GCR-HA, △433-510aa named △DBD-HA, △545-792aa named △LBD-HA) and three fragment plasmids were constructed. (**F**) The full-length STAT1 (STAT1-Flag) and protein binding domain truncation (△1-121aa named △STAT_int -Flag) and one fragment plasmid was constructed. (**G**) Extracts of HEK293T cells transfected with functional truncations of HA-GR plasmids and Flag-STAT1 plasmid were immunoprecipitated with HA antibodies. (**H**) Extracts of HEK293T cells transfected with functional truncation of Flag-STAT1 plasmids and HA-GR plasmid were immunoprecipitated with Flag antibodies. BMDMs, bone marrow-derived macrophage; DXM, dexamethosone; GR, glucocorticoids receptor; mGR, membrane glucocorticoids receptor; MTX, methotrexate; STAT1, signal transducer and activator of transcription.

Our previous study has demonstrated that GCs promote OC formation by binding to mGR.^13^ To further elucidate the mechanism underlying the regulation of DXM-induced osteoclastogenesis by MTX, we conducted mass spectrometry (MS) analysis using anti-GR antibodies in bone marrow-derived macrophages (BMDMs) treated with bovine serum albumin-conjugated DXM (DXM-BSA) in the presence or absence of MTX (figure 5A). Among several potential candidates, signal transducer and activator of transcription 1 (STAT1) was identified as a downregulated protein in the MS results in the in the D+M group compared with DXM group (online supplemental table 1). Moreover, MS analysis identified a peptide of STAT1, suggesting a possible interaction between GR and STAT1 (figure 5B). STAT1 has previously been shown to be a negative factor in OC formation.^24 25^ Therefore, we hypothesised that MTX reduced the interaction of GR and STAT1 to suppress OC differentiation. To confirm the protein from MS results, coimmunoprecipitation (Co-IP) and immunofluorescence assays were performed. These results indicated that DXM-BSA enhanced the interaction of GR with STAT1, while their interaction was weaker in BMDMs treated with DXM-BSA and MTX compared with that treated only with DXM-BSA (figure 5C,D). In order to map the regions of GR that interact with protein interaction domain of STAT1, we generated three functional truncations of GR protein, including GC receptor GCR (1-418aa), DNA-binding domain of GC receptor (DBD-GR, 433-510aa), ligand binding domain of the GC receptor (LBD-GR, 546-792aa) and truncation of protein interaction domain of STAT1 (2-121aa), respectively (figure 5E,F). And we found that a truncation of GR (△DBD-GR) and truncation of STAT1 protein binding domain, which both destroyed the interaction of GR and STAT1 (figure 5G,H). Altogether, these results strongly supported that the interaction and functional connection between GR and STAT1 in the presence of DXM-BSA are reduced by MTX.

### STAT1 is activated by MTX via enhancing the interaction of A2AR and IFN-γR

**Figure 6:**
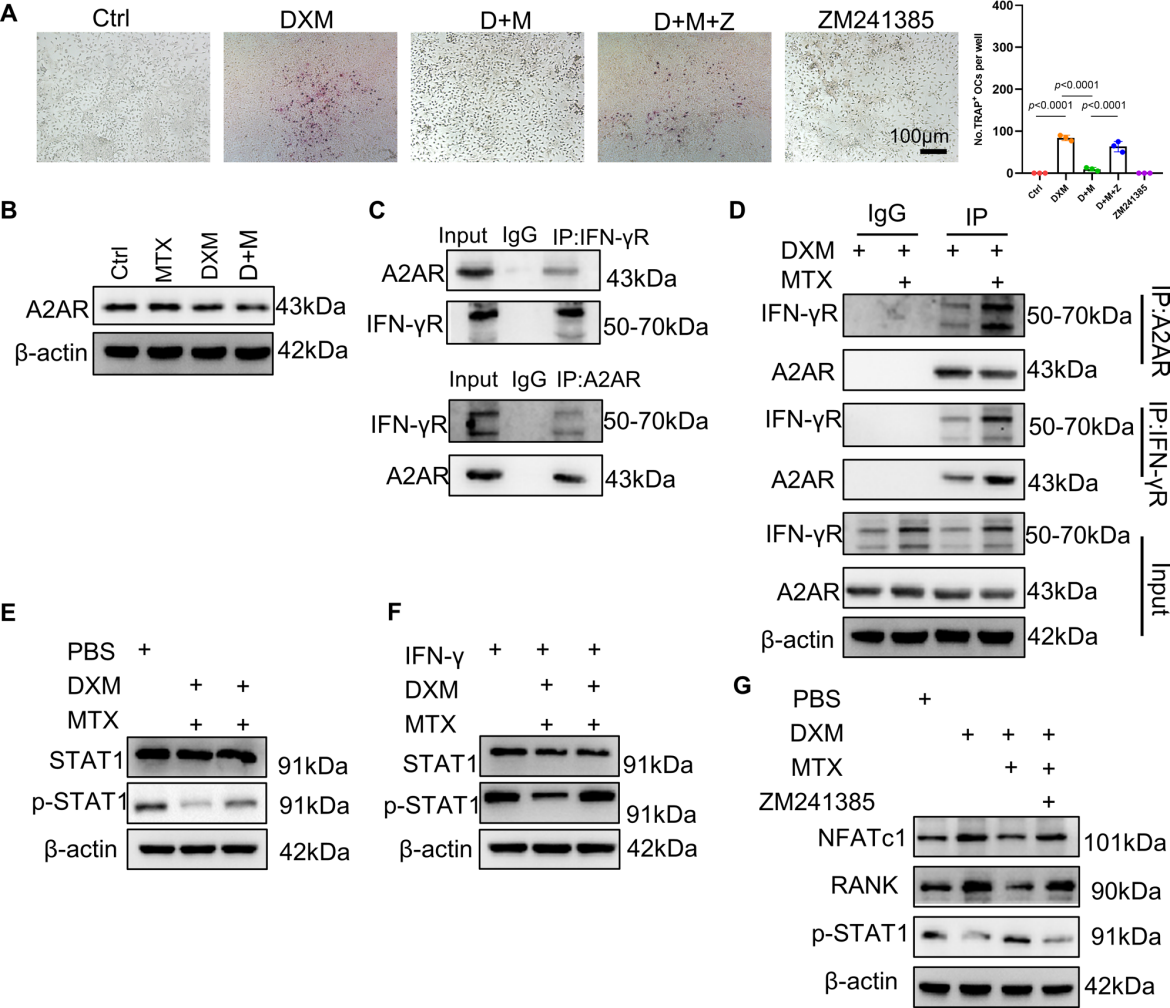
The mechanism of MTX reducing the interaction of membrane glucocorticoids receptor and STAT1. (**A**) Representative images and quantification of TRAP-positive osteoclasts differentiated from BMDMs in the presence of PBS (Ctrl), DXM, D+M, D+M+Z (ZM241385) and ZM241385 (a highly selective antagonist for A2AR); D represents DXM, M represents MTX, Z represents ZM241385; scale bar: 100 µm. (**B**) The A2AR protein level in BMDMs treated with bovine serum albumin-conjugated DXM (DXM-BSA), MTX or PBS detected by Western blotting (WB). (**C**) Extract of BMDMs was immunoprecipitated with IgG, A2AR and IFN-γR antibodies. (**D**) Extract of BMDMs treated with DXM-BSA or MTX for 24 hours was immunoprecipitated with IgG, A2AR and IFN-γR antibodies. (**E**) The protein level of p-STAT1 in BMDMs treated with DXM-BSA, MTX or PBS detected by WB. (**F**) The protein level of p-STAT1 induced by IFN-γ in BMDMs treated with DXM-BSA, MTX or PBS detected by WB. (**G**) The protein level of nuclear factor of activated T cells 1, RANK and p-STAT1 treated with DXM-BSA, MTX or PBS in the presence of A2AR inhibitor or not detected by WB. In (**A**), data were analysed using a one-way analysis of variance, post hoc Tukey’s test. Data are shown as means±SD from three separate experiments. BMDMs, bone marrow-derived macrophage; DXM, dexamethosone; MTX, methotrexate; STAT1, signal transducer and activator of transcription.

MTX is known to inhibit osteoclastogenesis through activation of adenosine A2A receptor (A2AR).^17^ Therefore, we wonder whether MTX abolish the DXM-mediated OC formation via A2AR. We observed the inhibitory effect of MTX on osteoclastogenesis was reversed by the addition of ZM241385 (figure 6A), a highly selective antagonist for A2AR.^26^ Thus, we speculated that MTX reduce the interaction of GR and STAT1 via A2AR to inhibit osteoclastogenesis, while we did not observe a direct interaction between A2AR and GR or STAT1 (online supplemental figure 2A). Additionally, we found that A2AR expression was not affected by DXM-BSA or MTX (figure 6B). We thus hypothesised that MTX reduced the interaction of GR and STAT1 via A2AR interacting with another protein. Given that STAT1 was activated in IFN-γR, we wonder whether there is an interaction between A2AR and IFN-γR. Co-IP confirmed A2AR and IFN-γR protein interacted with each other (figure 6C). In addition, the interaction of A2AR and IFN-γR protein was strengthened in the presence of MTX compared with that treated only with DXM-BSA (figure 6D).

Previous study revealed an inhibitory effect of IFN-γ on OC differentiation via IFN-γR/STAT1 signalling.^27^ We also observed a marked inhibitory effect of IFN-γ on OC formation in DXM-stimulated BMDMs (online supplemental figure 3A–B). Treatment with DXM-BSA, IFN-γR expression was reduced (online supplemental figure 4A–B). On the contrast, MTX took no action on IFN-γR expression, while MTX rescued the IFN-γR expression downregulated by DXM (online supplemental figure 4A–B). Besides, MTX exerted no role in STAT1 activation (online supplemental figure 4), but MTX abrogated a downward trend in the levels of phosphorylated STAT1 (p-STAT1) caused by DXM-BSA (online supplemental figure 4D, figure 6E,F). Above findings indicate that MTX activates IFN-γR/STAT1 signalling relying on the presence of DXM. To directly investigate the role of MTX in STAT1 activation, we used ZM241385 to inhibit the A2AR activation induced by MTX. Western blots analysis showed that DXM-induced osteoclastogenesis-related makers and DXM-suppressed STAT1 activation were neither rescued by MTX in the presence of ZM241385 (figure 6G), suggesting that MTX inhibits DXM-induced osteoclastogenesis through activating IFN-γR/STAT1 signalling via A2AR. Overall, MTX inhibits osteoclastogenesis by enhancing the interaction of A2AR and IFN-γR to activate IFN-γR/STAT1 signalling.

### STAT1 activation is critical for inhibition of DXM-induced osteoclastogenesis by MTX

**Figure 7:**
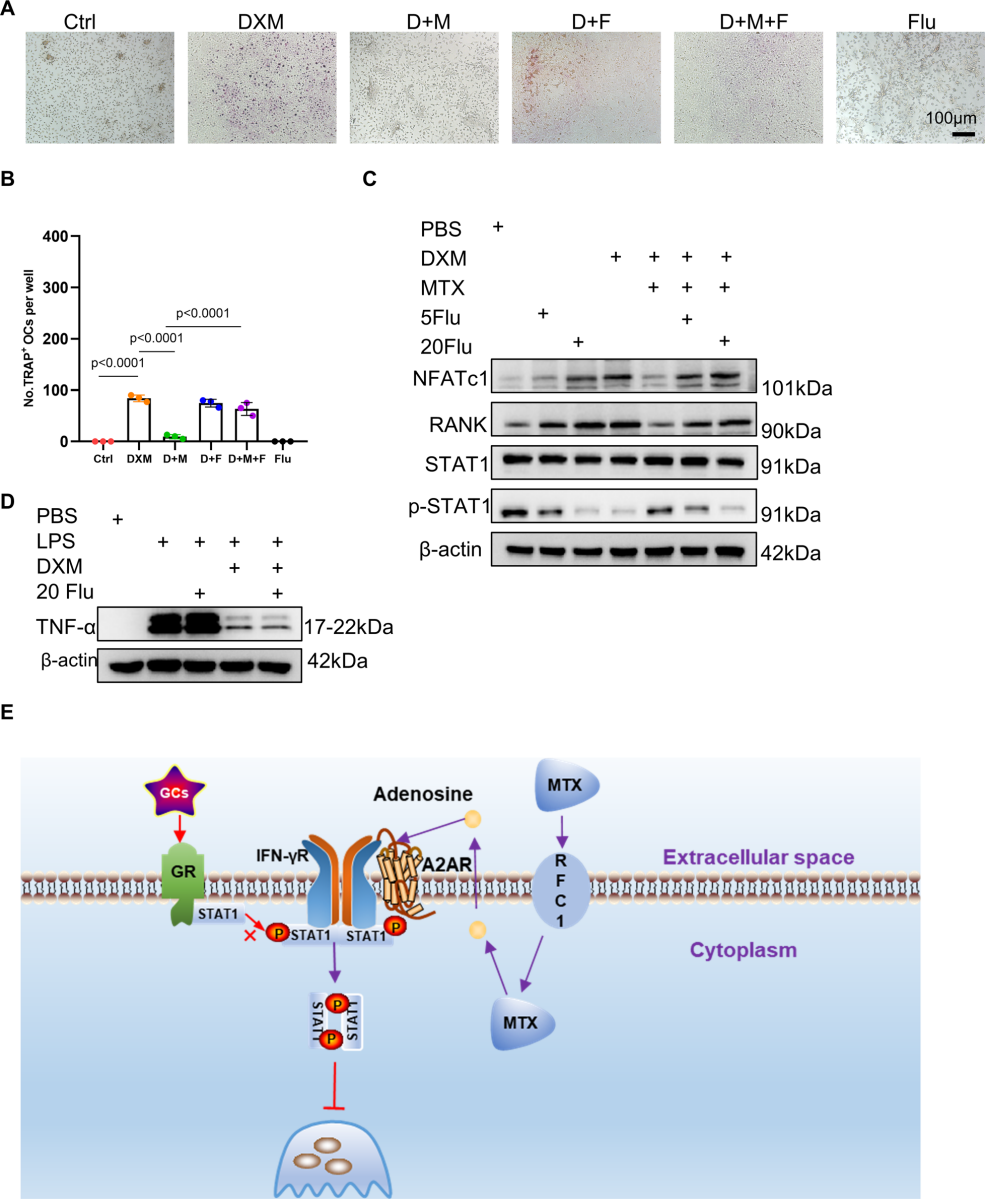
The effects of STAT1 activation on DXM function. (**A,B**) Representative images (**A**) and quantification (**B**) of TRAP-positive osteoclasts differentiated from BMDMs showing Ctrl, DXM, D+M, D+F, D+M+F and influenza (a specific STAT1 activation inhibitor); D represents DXM, M represents MTX, F represents fludarabine; scale bar: 100 µm. (**C**) The protein level of NFATc1, nuclear factor of activated T cells 1, RANK, STAT1 and p-STAT1 in BMDMs treated with DXM-BSA, MTX or PBS in the presence and absence of fludarabine or not detected by Western blotting. (**D**) The protein level of TNF-α in BMDMs treated with LPS, DXM, fludarabine or PBS. (**E**) Diagram of the proposed role of MTX in regulating osteoclast induced by GCs. In (**B**), data were analysed using a one-way analysis of variance, post hoc Tukey’s test. Data are shown as means±SD from three separate experiments. BMDMs, bone marrow-derived macrophage; DXM, dexamethosone; GCs, glucocorticoids; GR, GC receptor; LPS, lipopolysaccharide; MTX, methotrexate; STAT1, signal transducer and activator of transcription.

To validate the role of STAT1 activation in mediating the inhibitory effects of MTX on DXM-induced osteoclastogenesis, we used fludarabine, a specific IFN-γR/STAT1 pathway inhibitor,^28^ to block STAT1 activation and to determine whether it abolished the anti-osteoclastogenic activity of MTX. We found that fludarabine diminished inhibitory effect of MTX on DXM-induced osteoclastogenesis and did not affect DXM-induced osteoclastogenesis (figure 7A,B). Western blot results further revealed that fludarabine markedly upregulated osteoclastogenesis-related markers including NFATc1 and RANK downregulated by MTX in the presence of DXM, indicating that the anti-osteoclastogenic activity of MTX was abolished in response to fludarabine treatment (figure 7C). Besides, we wonder whether blocking STAT1 activation changes the anti-inflammatory effects of DXM. Western blot results showed that fludarabine did not affect TNF production induced by LPS and inhibitory effect of DXM on TNF production induced by LPS (figure 7D). These results indicate that blocking STAT1 activation does not affect anti-inflammatory effect of DXM

Collectively, these findings implied that MTX inhibits DXM-induced osteoclastogenesis through IFN-γR/STAT1 signalling pathway.

## Discussion

In the context of RA, the coadministration of GCs and MTX is crucial for early intervention following disease onset, particularly to address synovitis, while the molecular mechanisms underlying the role of MTX have not been fully elucidated. In the present study, we found that MTX does not exert significant effects on acute inflammation but should be combined with GCs to promptly reduce RA activity. We have discovered that MTX is ineffective in suppressing the release of TNF-α, yet it does exhibit the ability to inhibit OC activation induced by TNF-α (data not published). Importantly, MTX was found to mitigate bone damage induced by GCs during RA treatment. Mechanistic investigations demonstrated that MTX inhibits DXM-induced OC formation by enhancing the interaction between A2AR and IFN-γR, leading to reduced interaction between GR and STAT1, thereby activating the IFN-γR/STAT1 signalling pathway.

RA is a chronic systemic inflammatory disease affecting not only joints but also vital organs such as the heart, lungs, liver and kidneys.^29^ Due to their potent anti-inflammatory properties, GCs are preferred in clinical practice for managing RA-related inflammation. In our study, we also confirmed that GCs, rather than MTX exhibits strong anti-inflammatory effects both in vivo and in vitro in acute inflammation. GCs conduct anti-inflammatory effect through cytosolic GR binding, subsequent translocation of the GCs-GR complex to the nucleus, and modulation of anti-inflammatory or pro-inflammatory gene expression.^6 30 31^ In line with our findings, DXM induced GR nucleus translocation, while MTX plays no role in this process.

Recent studies have reported that the combined administration of MTX and low-dose GCs can slow down bone damage in patients with RA,^4 32^ although the specific underlying mechanism remains elusive. Notably, long-term GC use is associated with an increased risk of osteoporosis due to enhanced OC differentiation and maturation leading to bone damage,^33^ which limit the application of GCs. GCs mainly increase differentiation and maturation of OCs to induce bone damage,^20^ and current clinical treatments of osteoporosis, can help reduce bone loss but may pose risks such as increased breast cancer risk, thromboembolism and osteonecrosis of the jaw.^34^,^36^ Therefore, it is necessary for identifying novel therapeutic targets for GIOP. Our previous research demonstrated that GCs promote osteoclastogenesis through mGR by using DXM-BSA, a membrane-restricted GCR agonist.^13^ Another previous study demonstrated that GCs treatment decreases p-STAT1 expression (a key mediator of IFN-γ responsiveness) in mononuclear cells from multiple sclerosis patients.^37^ Moreover, other studies similarly reported that addition of DXM led to a decreased level of p-STAT1.^38 39^ More recently, Ballegeer *et al* verified that GR dimers repressed the phosphorylation state of STAT1 in intestinal epithelium cell.^40^ Above studies illustrated the mechanism of anti-inflammatory action GCs. Here, we also observed that DXM repressed p-STAT1 expression in the process of OC formation. We believe STAT1 was less recruited to be phosphorylated due to the increase of the interaction between mGR and STAT1 by using DXM-BSA. STAT1 activation is essential for responsiveness of IFN-γ,^41^ and we observed that IFN-γ obviously inhibited DXM-induced osteoclastogenesis, suggesting STAT1 is critical for GCs-mediated OC formation. MTX has been confirmed to inhibit OC formation in various studies.^9 17 18^ However, there is little evidence regarding MTX inhibiting GCs induced osteoclastogenesis. In this study, we provide evidence that MTX can suppress OC formation induced by GCs. We also revealed that MTX enhanced the interaction of A2AR and IFN-γR to activate IFN-γR/STAT1 signalling pathway. Thus, MTX decreased the interaction of mGR and STAT1 increased by DXM potentially due to increased recruitment of STAT1 to the IFN-γR binding site. However, the study failed to explore the underlying mechanism of MTX to regulate the interaction between A2AR and IFN-γR. Indeed, future studies are warranted to elucidate how MTX modulates the interaction between A2AR and IFN-γR.

In conclusion, our study sheds new light on the crucial role of MTX in inhibiting GC-induced osteoclastogenesis through the activation of the IFN-γR/STAT1 signalling pathway. These findings offer valuable insights into the combination therapy of MTX and GCs for RA treatment, particularly in the context of bone erosion and may hold clinical significance for optimising treatment strategies for RA-related bone damage.

## supplementary material

10.1136/rmdopen-2024-004886 online supplemental file 1

## Data Availability

Data are available upon reasonable request.
