# Supplementary material for: Methotrexate inhibits glucocorticoids-induced osteoclastogenesis via activating IFN-γR/STAT1 pathway in the treatment of rheumatoid arthritis
Source: RMD Open. 2024 Nov 7;10(4):e004886. doi: 10.1136/rmdopen-2024-004886 (PMC11552566; doi:10.1136/rmdopen-2024-004886)
Supplement: online supplemental file 1 [file rmdopen-10-4-s001.pdf]

Supplemental materials

Supplemental methods

Cell isolation, culture and activation and induction

Bone marrow-derived macrophages (BMDMs) were isolated from 6-8 weeks C57BL/6 mice. BMDMs were cultured in MEM-α (Gibico) medium supplemented fetal bovine serum (FBS) (Gibico), 1% penicillin–streptomycin and 25 ng/mL of M-CSF (Z02930, Genscript) in 5% CO2 at 37 °C. To induce osteoclast formation, BMDMs were treated with 10 μm dexamethasone (DXM) (D4902, Sigma-Aldrich) or 1 μm DXM-BSA recombinant 25ng/ml RANKL alongside M-CSF for 3 or 6 days to induce osteoclastogenesis, in the presence or the absence of 0.1, 2 or 5μm methotrexate (MTX) (HY-14519, MedChemexpress), with 20 μm fludarabine (FLU) (HY-B0069, MedChemexpress) or 1μm ZM241385 (HY-19532, MedChemexpress), with replacement of the culture medium every 3 days, then prepared for further experiments. Tartrate Resistant Acid Phosphatase (TRAP) staining was used to evaluate the osteoclasts following the TRAP kit (Cat. 387A, Sigma-Aldrich) protocol. To induce inflammation in vitro, BMDMs were treated with 100ng/ml Lipopolysaccharide (LPS) (L2630, Sigma-Aldrich) for 30 min or 6 h, with 1 μg/ml Brefeldin A (BFA) (HY-16592, MedChemexpress) to inhibit cytokines secretion, then the cells were harvested for protein extraction.

Mouse osteoblast precursor cells MC3T3-E1 was contributed by the Huazhong University of Science and Technology, Wuhan, China. MC3T3-E1 cells were cultured in MEM-α medium supplemented FBS and 1% penicillin–streptomycin. Then the

medium was removed, osteogenic medium containing β-glycerophosphate (10 mM), dexamethasone (10 nM), and ascorbic acid diphosphate (50 μg/ml) was added and continuously cultured for 7 days. On 7th day, Alkaline Phosphatase (ALP) Staining was performed to analyze the bone induction ability by using ALP staining kit (294–67001, WAKO).

Quantitative/Real-Time Polymerase Chain Reaction (qRT-PCR)

Total RNA was extracted from cells using TRIzol reagent (R411, Vazyme). GAPDH was used as an internal control for mRNA. cDNA was obtained by reverse transcription with using ABScript III RT Master Mix for qPCR with gDNA Remover (RK20429, Abclonal). qRT-PCR was performed using 2 × Universal Blue SYBR Green qPCR Master Mix (G3328-01, Servicebio). Relative expression was calculated using the $2^{-\Delta\Delta Ct}$ method. The primers used in this study were listed as follows: mouse TNF-α forward: 5’-CCCTCACACTCAGATCATCTT CT-3’, reverse: 5’-GCTACGACGTGG GCTACAG-3’; mouse NFATc1 forward: 5’-GACCCGGAGTTCGACTTCG-3’, reverse: 5’-TGACACTAGGGGACACATAACTG-3’); mouse RANK forward: 5’-GGACGGTGTTGCAGCAGAT-3’, reverse: 5’-GCAGTCTGAGTTCCAGTGGTA-3’; mouse GR forward: 5’-AGCTCCCCC TGG TAGAGAC-3’, reverse: 5’-GGT GAAGA CGCAGAAACCTTG-3’; mouse Balap2 forward: 5’-CTGACCTCACAGATC CCAAGC-3’, reverse: 5’-TGGTCTG ATAGCTCGTCACAAG-3’; mouse OPN forward: 5’-AGCAAGAAACTCTTCCAAGCAA-3’, reverse: 5’-GTGAGATTC GTCAGATTCATCCG-3’; mouse ALP forward: 5'-AC CTCCTCGGAAGACACT CTG-3', reverse: ACTGCGCCTGGTAGTTGTTG; mouse RUNX2 forward: 5’-

ATGCTTCATTCGCCTCACAAA-3’, reverse: 5’-GCACTCACTGACTCGGTTGG-3’; mouse β-actin forward: CTCCATCCTGGCCTCGCTGT, reverse: GCTGTCACCTTCACCGTTCC.

Transient transfection of plasmids

Plasmids were purchased from Wuhan Genecreate company. HEK 293T cells were seeded in plates, and cells then were transfected plasmids by using Lipofectamine 8000 reagent (C0533, Beyotime) according to the manufacturer's instructions.

Western blot and Co-IP

Nuclear and cytoplasmic protein was extracted using the Nuclear-Cytosol Extraction Kit (P1200, Applygen) according to the manufacturer’s instructions. BMDMs were lysed with RIPA buffer (G2002, Servicebio) or IP lysis buffer (G2038, Servicebio) supplemented with PMSF (G2008, Servicebio) and protease and phosphatase inhibitor cocktail (G2007, Servicebio). For Co-IP assay, primary antibody was added to the supernatant, and the samples were incubated overnight at 4°C. The next day, and Protein A/G magnetic beads (HY-K0202, MedChemexpress) was added to the supernatant and incubated for 4 hours. Then, the beads washed three times with IP lysis buffer. 1 × SDS-PAGE loading buffer was mixed with the beads, followed by heating at 95 °C for 10 min to elute beads for further immunoblotting. The extracted protein was immunoblotted with the primary antibodies using standard protocols. Protein bands were detected by ChemiDoc Touch Imaging System (Bio-Rad, CA, USA).

The primary antibodies include anti-phospho-NF-κB p65 (Ser536) (dilution 1:1000，3033, Cell Signaling Technology), anti-Phospho-IκBα (dilution 1:1000, TP56280F，

Abmart), anti-Phospho-p38 (dilution 1:1000, #4511, Cell Signaling Technology), anti-phospho-JNK (dilution 1:1000, 80024-1-RR, Proteintech), anti-Phospho-Erk (dilution 1:1000T40072, Abmart), anti-TNF-α (dilution 1:1000，26405-1-AP, Proteintech), anti-NFATc1 (dilution 1:1000, Selleck), anti-RANK (dilution 1:1000, A13382, Abclonal), anti-Glucocorticoid receptor (dilution 1:1000，24050-1-AP, Proteintech), anti-IFNγR (dilution 1:1000, 10808-1-AP, Proteintech), anti-STAT1 (#14994, Cell Signaling Technology), anti-Phospho-Stat1 (dilution 1:1000, #9167, Cell Signaling Technology), anti-A2AR (dilution 1:1000, A1587, Abclonal), anti-Histone H3 (1:2000, #BF9211, Affinity), anti-β-actin (dilution 1:2000, #8457, Cell Signaling Technology). The secondary antibodies include HRP-conjugated Affinipure Goat Anti-Mouse IgG (dilution 1:5000, SA00001-1, Proteintech), HRP-conjugated Affinipure Goat Anti-Rabbit (dilution 1:5000, SA00001-2, Proteintech), and Mouse Anti-rabbit IgG Conformation Specific (L27A9) mAb (dilution 1:2000, #3678, Cell Signaling Technology) used for CO-IP samples. β-Actin was used as the control for total and cytoplasm protein, Histone H3 was used as the control for nuclear protein.

Liquid chromatography tandem-mass spectrometry (LC-MS/MS) analysis

BMDMs treated with DXM-BSA in the presence of MTX or not for 24 h. BMDMs were harvested for immunoprecipitation using the GR antibodies. The protein identification via mass spectrometry (MS) was performed by Shanghai Zhongke New Life Biotechnology company.

Cell viability assay

BMDMs were seeded in 96-well plates at a concentration of 1 × 10^5 cells/mL, with

100 μL per well, and with 6 replicate wells for each group. The cytotoxic effects of MTX on BMDMs were investigated using a Cell Counting Kit-8 (CCK-8) assay. At 72 and 144 h, 10 μL of CCK-8 buffer (C0037, Beyotime) was added into the medium. After 1 h of incubation, the optical density (OD) was measured at a wavelength of 450 nm by using a microplate reader.

Flow cytometric analysis

Fluorochrome-conjugated antibodies (5ul per test) were used to stain BMDMs for 30 min, including FITC-conjugated anti-F4/80 (E-AB-F0995C, Elabscience) and PE-conjugated IFN gamma Receptor 1 (12-1191-82, Invitrogen). The stained cells were measured by FACScan flow cytometer (BD, USA).

Fuorescence immunocytochemical staining

BMDMs cells were seeded on confocal dish, and were treated with 4% formalin at room temperature for 15 mins, then cells were permeabilized in 0.5% Triton X-100 for 15 min at room temperature. or fluorescence immunocytochemical staining, the cells then were blocked with 5 % bovine serum albumin (BSA) for 1 h, which followed by incubating with a primary antibody overnight at 4 °C. After three washes in PBS, the cells were incubated with a secondary antibody for 1 h at room temperature in the dark. Last, the nuclei were stained with DAPI (D9542, Sigma-Aldrich). Images were acquired by the confocal fluorescence microscope. Primary antibodies include anti-Glucocorticoid receptor (dilution 1:200, 24050-1-AP, Proteintech), anti-Phospho-Stat1 (Ser727) (dilution 1:200, #8826, Cell Signaling Technology), anti-IFNγR (dilution 1:200, 10808-1-AP, Proteintech), anti-A2aR (dilution 1:50, A1587, Abclonal). Second

antibodies include CoraLite488-conjugated Goat Anti-Rabbit IgG(H+L) (dilution 1:200, RGAR002, Proteintech) and Cy3–conjugated Affinipure Goat Anti-Rabbit IgG(H+L) (dilution 1:200, SA00009-2, Proteintech).

Enzyme-linked immunosorbent assay (ELISA)

The levels of type I collagen cross-linked C-terminal peptide (CTX-1) and soluble RANKL in the mouse serum from the harvested GIOP mice on day 29, were measured using ELISA Kit (E-EL-M3023, Elabscience) and mouse soluble RANKL ELISA Kit (EE05127, CUSABIO), respectively.

Hematoxylinand Eosin Staining, Immunohistochemistry, TRAP staining and ALP staining of femurs

All femur specimens of mice were decalcified in EDTA decalcifying solution for 30 days before paraffin embedding after micro-CT examination. Next, these tissue sections were subjected to hematoxylin and eosin staining (H&E) and immunohistochemistry (IHC) as standard protocols. Arthritis inflammation was evaluated as previously reported (14, 15).

Femur sections were subjected to TRAP, and ALP staining using TRAP&ALP kit (294–67001, WAKO) following the manufacturer's protocols. Subsequently, nucleus was stained with 0.5% methyl green solution (G1670, Solarbio).

The IHC, TRAP and ALP staining intensity was scored in blinded fashion: 1= weak staining; 2 = medium staining; 3 = strong staining. The positive percentage was defined as follow: 0 = 0%, 1 = 1%–25%, 2 = 26%–50%, 3 = 51%–75%, 4 = above 75%.

Micro-CT analysis of femurs

Micro-CT (SkyScan 1176, Bruker) with Al 0.5 filter was used to analyze the microarchitecture of the femurs harvested from GIOP mice on day 29. Three-dimensional was reconstructed by by using NRecon (Bruker), and 3D model reconstruction image was analyzed using CTAn (Bruker).

Statistics

Statistical analysis was analyzed with GraphPad Prism9.3.0 software and presented as the means ± standard deviation (SD). Differences between the two groups were conducted through unpaired two-tailed Student's t-test (parametric) or one-way analysis of variance (ANOVA) for more than two groups**.** $p< 0.05$ was considered statistically significant.

Supplemental Figure and figure legends

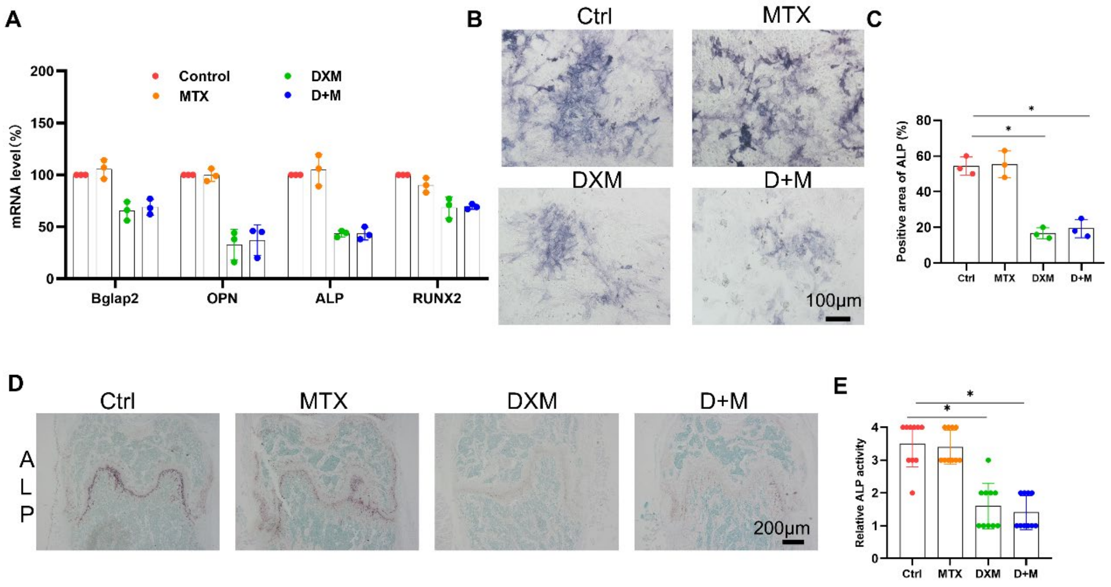

Supplemental Figure 1. The role of MTX in DXM-reduced osteoclast formation.

(A) The mRNA level of Bglap2, RNAK, OPN, ALP and RUNX2 in BMDMs treated with DXM, MTX, or PBS for 24h were evaluated by RT-qPCR. **(B-C)** Representative

images (B) and quantification (C) of ALP positive osteoblasts cultured with DXM and or MTX; Scale bar: 100 μm. **(D-E)** Representative images (D) and scores (E) of TRAP staining of the femur; Scale bar: 200 μm. Data are shown as means ± SD from two separate experiments. In (A), (C), (E), data were analyzed using a one-way ANOVA, post hoc Tukey's test.

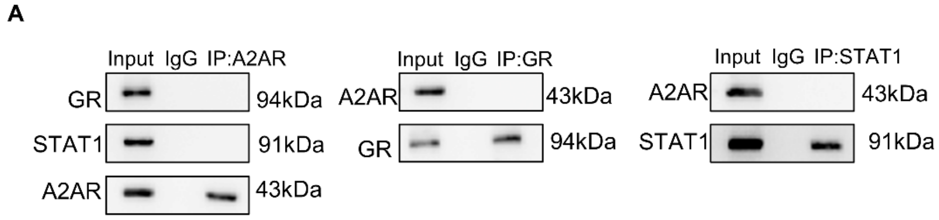

Supplemental Figure 2 The interaction of A2AR and GR or STAT1 in BMDMs. (A) Extract of BMDMs was immunoprecipitated with IgG, A2AR, GR and STAT1 antibodies.

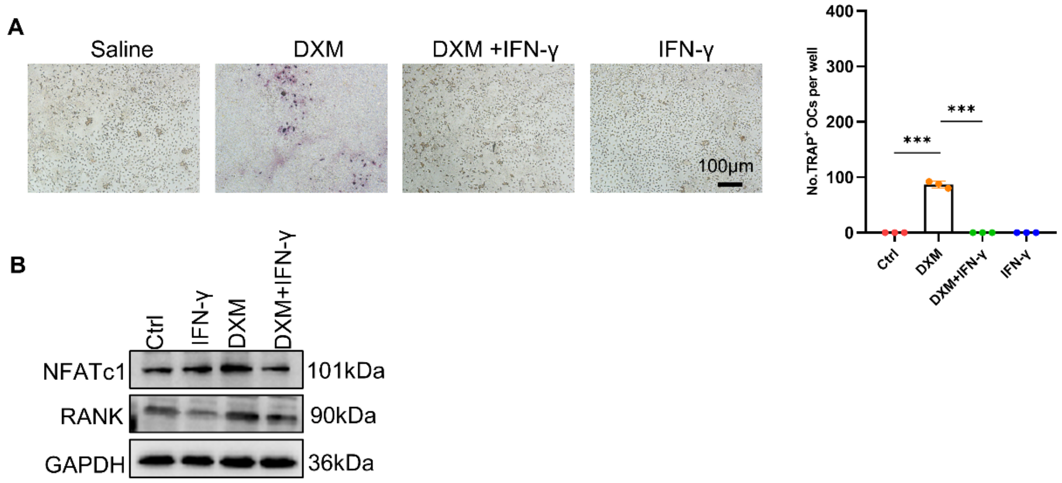

Supplemental Figure 3 The role of IFN-γ in DXM-induced osteoclastgenesis. (A) Representative images and quantification of TRAP positive osteoclasts differentiated

from BMDMs showing Ctrl, DXM, DXM + IFN-γ, IFN-γ. (B) The protein level of NFATc1 and RANK treated with DXM, IFN-γ, or PBS were detected by WB. In (A), data were analyzed using a one-way ANOVA, post hoc Tukey's test. Data are shown as means ± SD from three separate experiments.

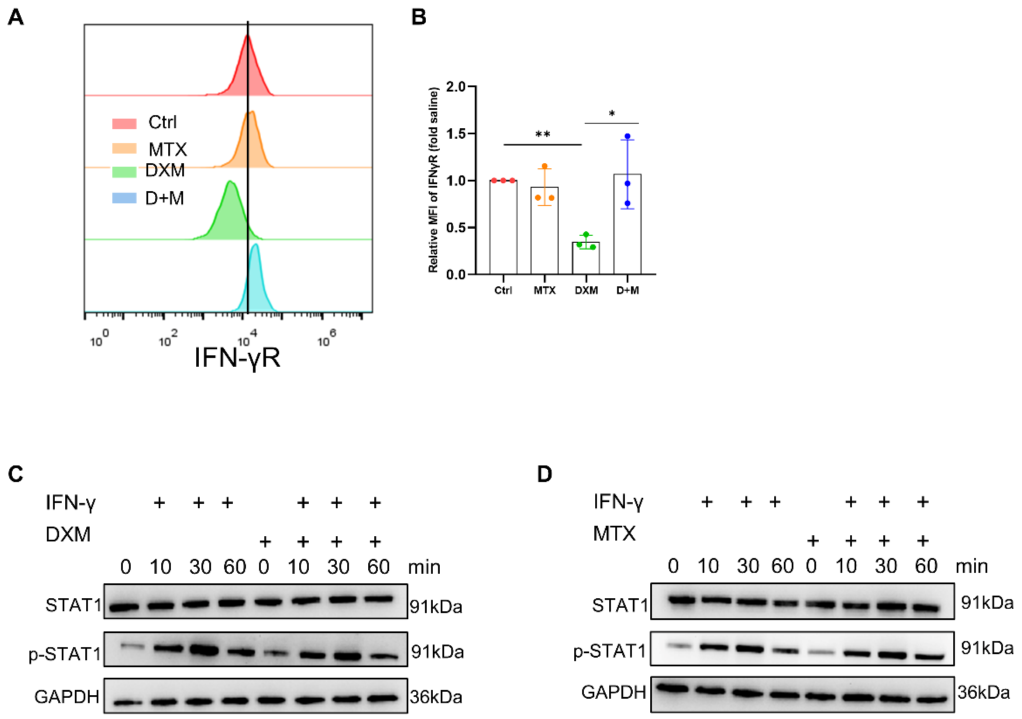

Supplemental Figure 4 The effects of MTX and DXM on IFN-γR/STAT1 activity. **(A-B)** The IFN-γR expression (A) and relative MFI (B) in BMDMs treated with DXM-BSA and/or MTX for 24 hours was evaluated by Flow cytometry. **(C)** The protein level of p-STAT1 induced by IFN-γ in BMDMs treated with DXM-BSA detected by WB. **(D)** The protein level of p-STAT1 induced by IFN-γ in BMDMs treated with MTX detected by WB. Data are shown as means ± SD from three separate experiments. In (B), data were analyzed using a one-way ANOVA, post hoc Tukey's test.

Supplemental table1

The mass spectrometry of glucocorticoid receptor

PROTEIN NAMES	IBAQ D	IBAQ D+M
**PROTEIN MAGO NASHI HOMOLOG 2**	1969100	0
**NADH-UBIQUINONE OXIDOREDUCTASE CHAIN 1**	0	147970000
**ZINC TRANSPORTER SLC39A7**	3712800	4820100
**TRANSCRIPTION ELONGATION FACTOR B POLYPEPTIDE 1**	1979700	0
**BAG FAMILY MOLECULAR CHAPERONE REGULATOR 2**	0	0
**VIGILIN**	336010	0
**PROTHYMOSIN ALPHA**	169330000	7287600
**ATP-DEPENDENT RNA HELICASE A**	3369400	247890
**ACTIN-RELATED PROTEIN 3**	8860000	0
**DYSTONIN**	0	9477800
**TAR DNA-BINDING PROTEIN 43**	1166200	0
**ELONGATION FACTOR 1-BETA**	6726600	0
**PROTEIN CDV3**	12999000	13160000
**SIGNAL TRANSDUCER AND ACTIVATOR OF TRANSCRIPTION 1**	222230000	18797000
